# Prevalence and Associated Factors of Post-COVID-19 Syndrome in a Brazilian Cohort after 3 and 6 Months of Hospital Discharge

**DOI:** 10.3390/ijerph20010848

**Published:** 2023-01-02

**Authors:** Juliana Lapa, Davi Rosa, João Pedro Lima Mendes, Rodolfo Deusdará, Gustavo Adolfo Sierra Romero

**Affiliations:** 1 Núcleo de Medicina Tropical, Faculdade de Medicina, Universidade de Brasília, Campus Universitário Darcy Ribeiro, Asa Norte, Brasília 70910-900, Brazil; 2 Instituto de Avaliação de Tecnologia em Saúde, Ramiro Barcelos Street, 2350, Santa Cecília, Porto Alegre, RS, Brasília 90035-903, Brazil

**Keywords:** long-COVID, post-COVID-19 syndrome, post-acute COVID-19, COVID-19

## Abstract

(1) Objectives: To evaluate the frequency and factors associated with the Post-COVID-19 Syndrome (PCS) in COVID-19 survivors after 3 and 6 months of hospital discharge; (2) Methods: We conducted a cohort study with patients who were hospitalized with COVID-19 in a referral public hospital in Brasília, Federal District, Brazil. After 3 and 6 months of discharge, patients answered a questionnaire about PCS symptoms. Poisson regression with robust variance was used to estimate the crude and adjusted prevalence ratios (PR and aPR) of PCS. (3) Results: The prevalence of PCS was 81% and 61% after 3 and 6 months of hospital discharge, respectively. The main symptoms after 3 months of discharge were hair loss (44%), fatigue (42%), and memory loss (39%); while after 6 months, they were memory loss (29%) and fatigue (27%). In the multivariate analysis, the main factor associated with PCS was female gender (aPR): 1.28 (1.16–1.41) and 1.60 (1.34–1.90), 3 and 6 months after hospital discharge, respectively. Hypercholesterolemia was also associated with PCS after 3 months aPR of 1.15 (1.04–1.27). After 6 months of discharge, obesity [aPR: 1.22 (1.03–1.45)] and pronation [aPR: 1.15 (1.06–1.25)] were relevant associated factors. (4) Conclusions: The prevalence of PCS was high in COVID-19 survivors who had the moderate and severe forms of the disease. Memory loss was the most persistent symptom. Our data pointed to female gender, hypercholesterolemia, obesity, and pronation during hospitalization as relevant PCS-associated risk factors.

## 1. Introduction

In December 2019, in Wuhan, China, a novel coronavirus, named SARS-CoV-2, was first detected, later spreading as an emerging respiratory pathogen that caused the COVID-19 syndrome [1]. In Brazil, the first case occurred on 25 February 2020, with a subsequent epidemic spreading over the entire country [2]. Currently, Brazil is the third-highest country in terms of cases of COVID-19, exceeding 30 million cases and more than 600,000 deaths. On the other hand, Brazil accumulated more than 29 million recovered patients [3].

Although many patients recovered from SARS-CoV-2 infection, they carried many types of sequelae during the follow-up. The National Institute for Health and Care Excellence (NICE) defined Post-COVID-19 Syndrome (PCS) as signs and symptoms that develop during or after an infection consistent with COVID-19, continue for more than 12 weeks, and are not explained by an alternative diagnosis [4].

According to a meta-analysis study, the prevalence of PCS ranged from 22% to 81% among studies addressed to hospitalized patients [5]. This high variability is related to the length of the follow-up, the hospitalization requirements, the definition of the disease, and the region where the study is conducted [6,7,8]. Another study points out the fact that the prevalence of PCS tends to decrease after 6 months [9].

Regarding risk factors for PCS, knowledge is still under construction. Risk factors such as female gender, older age, and the presence of comorbidities are very consistent in the literature [10,11,12]. However, the impact of other factors such as obesity, the severity of COVID-19 infection, and the requirement of oxygen, for example, is not clear yet [13,14,15,16,17]. The differences in the literature could be explained by regionalism, the period of the pandemic, and the characteristics of the studies. Studies in a developing country, during a different period of the pandemic, are important to access different prevalence ratios and factors associated with PSC.

The present study aims to estimate the frequency of PCS as well as its associated factors, following the NICE definition. For that, we considered COVID-19 survivors who had moderate and severe forms of the disease, evaluated 3 and 6 months after hospital discharge.

## 2. Materials and Methods

### 2.1. Study Design

A cohort study was conducted, including patients who were hospitalized with moderate or severe COVID-19 in a referral public hospital in Brasília, Federal District, Brazil.

The Regional Hospital of Asa Norte (HRAN) is a tertiary level hospital. In 2020, it was the main reference for COVID-19 for immunocompetent patients in the Federal District. At that time, the unit had 66 dedicated beds for COVID-19 patients, six of which were for critical care. The hospital offered quantitative reverse-transcription polymerase chain reaction for the diagnosis of COVID-19 (BiOMOL OneStep/COVID-19 IBMP, Allplex 2019-nCoV Assay Seegen, and molecular SARS-CoV2 (E/RP) Bio-Manguinhos) [18], computed tomography, and ventilatory assistance to patients who developed respiratory failure.

### 2.2. Sample Size

The sample was composed of all the patients who fulfilled the eligibility criteria during the predefined inclusion period from August through November of 2020.

### 2.3. Participant Eligibility

Inclusion criteria were age ≥ 18 years, informed consent from the patient or a responsible family member, a positive nasopharyngeal swab RT-qPCR for COVID-19, and a hospitalization requirement. Excluded from the study were patients who died during the hospitalization, those whom we could not contact by telephone, and patients or relatives unable to answer the questionnaires.

### 2.4. Outcomes

The primary outcome was the presence of PCS, defined as signs and symptoms that develop during or after an infection consistent with COVID-19, continue for more than 12 weeks, and are not explained by an alternative diagnosis.

The frequency of symptoms of PCS and the proportion of patients who needed support after discharge were evaluated.

### 2.5. Independent Variables

The independent variables that could be related to the outcome included social and demographic characteristics (age, sex, race (white x non-white), educational status (<1 year, ≥1 year and <8 years, ≥8 years and <12 years, ≥12 years of schooling), individual income, and comorbidities (hypertension, diabetes, obesity, hypercholesterolemia, and hypothyroidism)).

The characteristics of patients at hospital admission involved specific symptoms (e.g., cough, dyspnea, fever, myalgia, fatigue, headache, diarrhea, anosmia, chest pain, ageusia, nausea, vomiting, rhinorrhea, throat pain, anorexia/hyperoxia, and sneeze), median oxygen saturation, pulmonary involvement on tomography, and the level of severity based on the World Health Organization COVID-19 Scale.

By duration of hospitalization, we considered the length of hospitalization from the time of admission until discharge in terms of days. The treatment received included supplemental oxygen, invasive mechanical ventilation, the application of the prone position, and the use of antibiotics.

Data from hospitalizations was extracted from electronic medical records. The comorbidities were extracted based on a medical registration problem list at the time of admission. Obesity was defined as body mass index (BMI) ≥ 30 kg/m^2^ (weight in kilograms divided by the square of height in meters). The comorbidities (such as hypertension, diabetes, obesity, hypercholesterolemia, and hypothyroidism) were defined as dichotomous variables (yes/no). Sociodemographic information was collected from both medical records and interviews conducted by telephone.

In the analysis, the severity was stratified in three groups (mild, moderate, and severe): (1) mild: patients requiring hospitalization but no oxygen therapy (WHO scale—4 points); (2) moderate: patients requiring hospitalization and oxygen by mask or nasal prongs (WHO scale—5 points); and (3) severe: patients hospitalized requiring oxygen by non-invasive ventilation (NIV), high flow, intubation, and/or vasopressor support (WHO scale—6, 7, 8, and 9 points).

### 2.6. Follow-Up Interview after 3 and 6 Months

A telephone contact was made with eligible patients or their relatives, and, at that point, they were invited to participate in the study. The patients answered a questionnaire about PCS symptoms. Demographic characteristics, comorbidities, and other information not available in medical records were also included in the questionnaire.

The post-COVID symptoms investigated were intermittent fever, cutaneous signs, anosmia, ageusia, palpitation, cough, headache, chest pain, sleep disorders, depression, attention disorders, muscle pain, dyspnea, joint pain, memory loss, fatigue, and hair loss.

### 2.7. Statistical Analysis

Categorical data were expressed in frequencies and percentages; continuous data were reported as mean and standard deviation. For non-normal distribution data, medians and interquartile range (IQR) were used.

Categorical variables were compared using the chi-square test. In order to evaluate the factors associated with PCS and subgroups of specific symptoms, we divided them in five groups: pain (joint pain and muscle pain), fatigue (fatigue and dyspnea), dermatologic signs (hair loss and cutaneous signs), neurologic and psychiatric symptoms (headache, sleep disorders, depression, attention disorders, memory loss), and alteration in the senses (anosmia and ageusia). Poisson Regression with Robust Variance was used to estimate the crude and adjusted prevalence ratios with the respective 95% confidence intervals (95% CI) for the factors associated with PCS 3 and 6 months after hospital discharge. Variables at least moderately associated (*p* < 0.20) with the outcome in the bivariate analysis were selected for the multivariate analysis. A forward-looking stepwise regression approach was used. Also, in the final model, we decided to retain the variables age, sex, educational status, and individual income, irrespective of the *p*-value associated with them in the bivariate analysis.

## 3. Results

**Figure 1:**
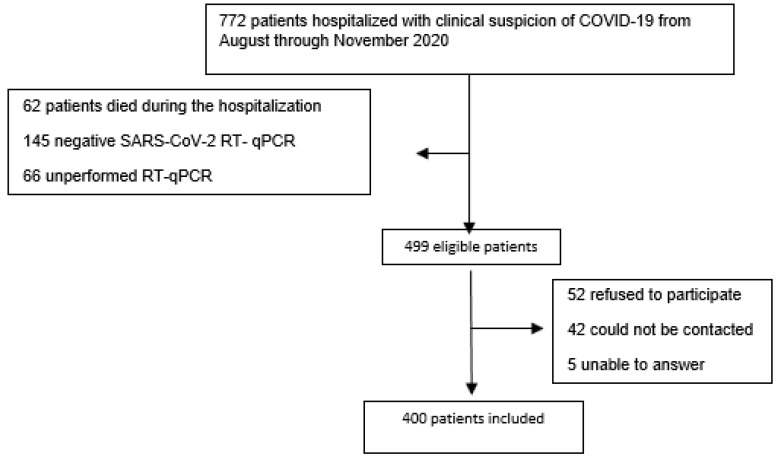
Flow chart of patients hospitalized with clinical suspicion of COVID 19 from August through November of 2020 in a referral public hospital in Brasília-DF—Brazil.

During the period from August through November of 2020, 772 patients with suspected symptomatic COVID-19 were hospitalized. We excluded 62 patients who died during the hospitalization, 145 who had a COVID RT-qPCR negative result, 66 who had not been submitted to RT-qPCR, 52 who refused participation, 42 whom we could not contact, and 5 patients who were unable to answer due to cognitive impairment and had no relatives to do it for them (Figure 1).

Then, a total of 400 patients were effectively enrolled in the study (Table 1). Out of that total, 51.6% were male and had a median age of 57 years, 63.8% were non-white, 35.1% were white, 78% had 12 years or less of schooling, and 52.8% were economically active before hospitalization. All of them were discharged after a maximum of 30 days after admission. During the hospitalization, 361 were admitted in an Emergency Department, 20 in a General Ward, five in an Intensive Care Unit. Seventy-two percent of the patients had some comorbidity before hospitalization. The most frequent ones were hypertension (68.1%), diabetes mellitus (39%), obesity (19%), hypercholesterolemia (15%), and hypothyroidism (10%).

Among the symptoms at admission (Table 2), the most common ones were cough (66%), dyspnea (66%), fever (51.6%), myalgia (35%), headache (29.6%), and diarrhea (22%). The median oxygen saturation at admission was 92% (IQR: 90–95), and most patients had pulmonary involvement between 25–75%.

The median hospital length was 8 (IQR: 6–13) days, nine patients were submitted to invasive ventilation, three developed stroke, six had a suspected pulmonary thromboembolic event, one was a confirmed case by computed tomographic pulmonary angiography, and five were not submitted to any confirmatory diagnostic method.

After 3 months, 81% of patients were experiencing at least one symptom associated with PCS. The median and the interquartile range number of symptoms was three (1–6). After 6 months, 61% had at a least one symptom, with median (IQR): 1 (0–3) (Table 3).

**Figure 2:**
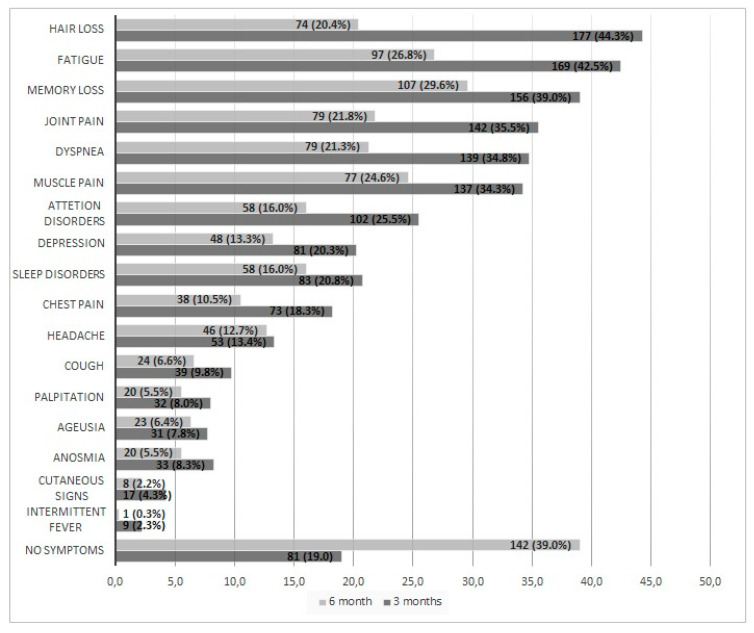
Frequency of symptoms of the Post-COVID Syndrome, considering 400 patients, 3 and 6 months after hospital discharge from a tertiary level hospital in Brasília, Federal District, Brazil, from August through November of 2020.

Symptoms after 3 months were: hair loss (44%), fatigue (42%), memory loss (39%), joint pain (36%), dyspnea (35%), muscle pain (34%), attention disorder (25%), depression (20%), and sleep abnormalities (20%) (Figure 2).

After 6 months, the most common symptoms were: memory loss (29%), fatigue (27%), muscle pain (24%), joint pain (22%), dyspnea (22%), and hair loss (20%) (Figure 2).

In the multivariate analysis, the main factor associated with PCS was female gender, with an adjusted prevalence ratio (aPR): 1.28 (95% CI: 1.16–1.41) and 1.60 (95% CI: 1.34–1.90), 3 and 6 months after hospital discharge, respectively (Table 4). Female gender was associated with a higher prevalence of pain [3 months: aPR 1.40 (95% CI: 1.11–1.79); 6 months: 2.17 (95% CI: 1.50–3.13) ], dermatologic signs [3 months aPR: 3.75 (95% CI: 2.82–4.99); 6 months: 3.80 (95% CI: 2.22–6.49)], and neuropsychiatric symptoms [3 months: aPR: 1.46 (95% CI: 1.21–1.75); 6 months: 1.55 (95% CI: 1.19–2.01)]. Female gender was associated with fatigue 6 months after hospital discharge (aPR: 1.45, 95% CI: 1.05–2.03). There was no significant association of female gender with fatigue 3 months after discharge (aPR: 1.15, 95% CI: 0.93–1.44) and sense alteration after 3 and 6 months with aPR of 1.17 (95% CI: 0.64–2.13) and 1.39 (95% CI: 0.66–2.92), respectively (Supplementary).

Among the comorbidities evaluated (hypertension, diabetes, obesity, hypercholesterolemia, and hypothyroidism), we did not find an association with PCS after multiple adjustments, except for obesity after 6 months. We also did not find any association between PCS and the use of supplemental oxygen, length of hospitalization, or severity (Table 4).

The multivariate analysis 3 months after discharge revealed an association of PCS with hypercholesterolemia (aPR: 1.15, 95% CI: 1.04–1.27) and, after 6 months, with obesity (aPR: 1.22, 95% CI: 1.03–1.45), and with the prone position (aPR: 1.15; 95% CI: 1.06–1.25) (Table 4).

## 4. Discussion

We found a high prevalence of PCS 3 and 6 months (81% and 61%) after hospital discharge in a cohort of patients who survived moderate-to-severe COVID-19 during the first pandemic wave. Three months after discharge, the main symptoms were hair loss (44%), fatigue (42%), and memory loss (39%), while memory loss (29%) and fatigue (27%) were the most frequent symptoms after 6 months.

A meta-analysis study that reviewed 15,577 studies from hospitalized and non-hospitalized patients found a prevalence of PCS of 45.9% (95% CI: 28.2–64.7) after 3 months. However, there is a high heterogeneity in these data I^2^: 96% [19]. Another meta-analysis study that included studies ranging from 14 to 110 days post-COVID infection estimated a prevalence of 80% of long-term symptoms [20]. Brazilian reports conducted in different regions found a prevalence of PCS higher than 80% suggesting that there may be a specificity of COVID-19 disease in Brazil [2,21,22].

The frequency of symptoms in our study was very similar to other studies, except for hair loss, which was much higher. In the literature, the most frequent symptom is fatigue (38.4%), with 95% CI of 30.4 to 47.4. Other authors have reported prevalences between 31% and 63% after 4 to 6 months [8,15,23].

The most long-lasting symptom was memory loss. Indeed, many studies showed that neurologic disturbance and fatigue were the most persistent symptoms. A meta-analysis study found a prevalence of memory loss/memory complaints/forgetfulness, and concentration difficulties of 19% after one year of follow-up [24].

The risk factors for the occurrence of PCS are unclear. Our exploratory analysis of factors revealed a significant association between PCS and female gender. Although it was a low-strength association (aPR of 1.60), it is consistent with the literature. It is also worth noting that female gender was associated with most of the subgroups of symptoms 3 or 6 months after hospital discharge.

It is not clear why women are more likely to develop PCS. Findings indicated that women develop stronger humoral and cellular responses to COVID-19 [25,26], which could perpetuate manifestations of symptoms and trigger autoimmune diseases. Indeed, some studies showed higher levels of autoantibodies and autoantigens in patients with COVID-19 [27,28]. It is also relevant to mention that the Myalgic Encephalomyelitis/Chronic Fatigue Syndrome syndrome, one possible mechanism of PCS, is 75% more prevalent in females [29].

The only group of symptoms that were not related to the female gender, neither after 3 nor after 6 months of discharge, was the alteration in the senses (anosmia and ageusia). Another study that evaluated the prevalence of those symptoms found that men were twice as likely to develop that kind of symptom [30]. Moreover, a Brazilian report that looked for factors associated with olfactory dysfunction did not find an association with gender [31].

We also found an association between hypercholesterolemia and PCS after 3 months (aPR:1.15, 95% CI: 1.04–1.27). In a previous study conducted in Germany, metabolic disorders were associated with long COVID outcomes [32]. Furthermore, hypercholesterolemia can increase a person’s susceptibility to SARS-CoV-2 and the risk of death from COVID-19 [3].

Obesity was associated with PCS (aPR 1.22; 95% CI: 1.03–1.45). According to the literature, obesity was associated with a higher incidence of PSC after seven months of hospital discharge [33]. This could be explained by the pro-inflammatory, immune, metabolic, and hormonal modifications due to obesity [33,34].

We found an association between PCS and prone position (aPR: 1.15; 95% CI: 1.06–1.25). Supposedly, this association is due to the severity of the disease in patients who required this measure. Our study failed to find a direct association between disease severity and PCS, although the evaluation 3 months after discharge showed point risk estimations with a discrete dose-response effect. A possible explanation would be that we studied a cohort of survivors underpowered for the detection of such a small effect and an underrepresented group of severe disease survivors.

The association between PCS and severity is widely discussed in the literature. Considering the physiopathology of the acute infection, an association between disease severity and the probability of having PCS would be expected. On the other hand, many articles showed a high prevalence of PCS in patients with mild symptoms. Bell et al. (2021) conducted a cohort study with non-hospitalized patients with COVID-19, and the prevalence of PCS was 77.1% [35]. The literature demonstrates the limitations of prediction models on this topic. Goërtz et al. demonstrated that even a model considering different variables predicted only 36% of the variance in PCS [36]. This demonstrates the urgency of further studies on this topic.

Our study has some limitations. It was a single-center study with a limited sample size, which restricted the generalizability of the results. On the other hand, the single-center approach ensures uniformity in data collection. The recruitment of participants was conducted by telephone, which could cause a selection bias. We had a follow-up loss of 38 patients (9.5%) from 3 to 6 months of interviews, which may have had an impact on the frequency estimates of events in the second interview. The lack of validated surveys to access post-COVID symptoms and the changes in the definition of this syndrome could have impacted the comparability of studies, though this is challenging for any study with COVID-19.

The prevalence of symptoms was based on self-reports, which is susceptible to information bias and misclassification. The evaluation of comorbidities (hypertension, diabetes, obesity, hypercholesterolemia, and hypothyroidism) was based on the medical registry, which is also vulnerable to misclassification. As we worked only with hospitalized patients, those results cannot be extrapolated to patients who had mild symptoms. Our data collection happened in the second semester of 2020, when the predominant variants were Gamma (Variant of Concern) and other variants non-Variants of Concern/ non-Variants of Interest (Zeta, B1, C.9, C.28, and C.33) [37]. Other variants could have a different impact on PCS. It is important to emphasize that there was no vaccination for COVID-19 during that period. Therefore, it is not possible to explore the effects of vaccination. That will need to be addressed in new cohorts of vaccinated individuals.

Notwithstanding the many studies reporting the prevalence of PCS, there are some specificities in the Brazilian COVID-19 epidemic. Brazil is a developing country, and it is the third-highest country in the world in terms of COVID-19 cases and deaths. Some variants circulated predominantly in Brazil [37]. At the time this study was conducted, there was no vaccine or specific treatment available for COVID-19. Our study had the strength of having accessed the same patients twice, which allowed for measuring the decrease in symptoms. Our findings reinforce the relationship between female gender and PSC, which is very consistent with previous literature. We also found a correlation between obesity and hypercholesterolemia with PSC. These associations are also described in the literature. Our lack of association of anosmia and ageusia with the female gender is also interesting. Many studies failed to find this association.

Finally, a global report conducted by Chen Chen et al. showed a 20% difference in the prevalence of PCS between continents [5]. Therefore, regional information is essential for knowledge and data comparison.

## 5. Conclusions

Our study confirmed the high prevalence of PCS after 3 and 6 months of discharge. Memory loss was the most persistent symptom. Female gender in 3 and 6 months, hypercholesterolemia in 3 months, obesity, and pronation in 6 months were associated with the syndrome.

Further studies should include a longer follow-up in order to assess the long-term impact and, as a consequence, the quality of life of the target population.

## Figures and Tables

**Table 1:** Baseline characteristics of 400 patients with moderate or severe COVID-19 admitted to a tertiary level hospital in Brasilia, Federal District in 2020.

Variables	n = 400	(%)
Male ^b^	207	51.8
Age ^b^	57 ^a^	46–67 ^a^
Race ^c,d^		
Non-white	255	63.8
White	140	35
Educational status (years of schooling) ^c^		
<1 year	22	5.5
≥1 year and <8 years	109	27.3
≥8 years and <12 years	64	16
≥12 years	184	46
Individual income ^e,f^ (R$)	1500	(1000–3000)
Comorbidities ^b^	290	72.5
Hypertension ^b^	196	67.8
Diabetes ^b^	113	39.1
Obesity ^b^	57	19.7
Hypercholesterolemia ^b^	43	14.9
Hypothyroidism ^b^	29	10.0
Current or former smoker ^b^	133	33.2

^a^ Median (interquartile range); ^b^ Data from electronic health records; ^c^ Self-report data; ^d^ Four patients did not answer, One with missing data; ^e^ 30 missing data; ^f^ Median (interquartile range).

**Table 2:** Frequency of symptoms at hospital admission, treatment, and complications during hospitalization of patients with COVID-19 admitted to a tertiary level hospital in Brasilia, Federal District, 2020.

	n = 400	%
Symptoms		
Cough	263	66.4
Dyspnea	253	63.9
Fever	230	57.6
Myalgia	138	34.8
Fatigue	135	33.6
Headache	117	29.5
Diarrhea	88	22.2
Anosmia	80	20.2
Chest pain	68	17.2
Ageusia	66	16.7
Nausea	47	11.9
Vomiting	42	10.6
Rhinorrhea	41	10.4
Throat pain	24	6.1
Anorexia/hyperoxia	23	5.7
Sneeze	2	0.5
Median oxygen saturation at admission ^a^	92	90–95
Pulmonary involvement on tomography		
<25%	65	17.2
25–50%	211	55.8
50–75%	93	24.6
>75%	9	2.4
Required supplemental oxygen	344	85.5
Invasive mechanical ventilation	9	2.25
Stroke	3	0.9
Length of hospital stay, days ^a^	8	6–13
Prone position		
Not Applied	270	67.3
Awake	128	32
Intubated	2	0.5
WHO COVID-19 scale ^b^		
4	56	14.0
5	253	63.2
6, 7, 8, 9	91	22.8

^a^ Median (interquartile range); ^b^ WHO scale: four patients requiring hospitalization but no oxygen therapy; five patients requiring hospitalization and oxygen by mask or nasal prongs; 6, 7, 8, and 9 patients hospitalized requiring oxygen by non-invasive ventilation (NIV), high flow, intubation, and/or vasopressor support, respectively.

**Table 3:** Number of symptoms 3 and 6 months after discharge of 400 patients with COVID-19 admitted to a tertiary level hospital in Brasilia, Federal District in 2020.

	3 Months	%	6 Months ^a^	%
0	77	19.3	142	39.2
1	54	13.5	82	20.5
2	43	10.8	40	10.0
3	39	9.8	38	9.5
4	45	11.2	27	6.8
5	34	8.5	22	5.5
6	31	7.7	15	3.7
7	21	5.3	12	3.0
8	19	4.7	9	2.2
9	9	2.3	6	2.5
10	12	3.0	3	0.8
11	7	1.8	1	0.2
12	6	1.5	1	0.3
13	2	0.5	2	0.5
14	1	0.2	0	0
15	0	0	0	0
16	0	0	0	0
17	0	0	0	0

^a^ Follow-up loss of 38 patients.

**Table 4:** Crude and adjusted prevalence ratios (PRR) between risk factors and Post-COVID Syndrome in a 3 and 6-month follow-up considering 400 patients in a tertiary level hospital in Brasilia, Federal District in 2020.

		3 Months				6 Months ^e^				
	n	PCS	PR	PRR ^a^ (95% CI)	aPRR ^a,b,c^ (95% CI)	n	PCS	PR	PRR ^a^ (95% CI)	aPRR ^a,b,d^ (95% CI)
Total	400	323	0.81			362	220	0.61		
Age										
≥57 years	199	164	0.82	1.04 (0.94–1.14)	0.99 (0.89–1.11)	180	113	0.63	1.07 (0.90–1.26)	1.02 (0.86–1.20)
<57 years	201	159	0.79	Ref	Ref	182	107	0.59	Ref	Ref
Sex										
Female	193	177	0.92	1.3 (1.18–1.43)	1.28 (1.16–1.41)	171	130	0.76	1.61 (1.36–1.92)	1.60 (1.34–1.90)
Male	207	146	0.70	Ref	Ref	191	90	0.47	Ref	Ref
Race (White x non-white)										
Non-white	260	210	0.81	1.00 (0.91–1.11)	0.98 (0.88–1.08)	240	144	0.60	0.96 (0.81–1.14)	0.95 (0.80–1.12)
White	140	113	0.81	Ref	Ref	122	76	0.62	Ref	Ref
Educational status (years of schooling)										
≥12 years	204	166	0.80	1.02 (0.92–1.12)	1.11 (1.00–1.24)	186	113	0.61	1.00 (0.85–1.18)	1.06 (0.89–1.26)
<12 years	196	157	0.81	Ref	Ref	176	107	0.61	Ref	Ref
Individual income										
≤R$ 1500	183	157	0.86	1.12 (1.02–1.23)	1.00 (0.99–1.00)	166	109	0.65	1.16 (0.98–1.37)	1.09 (0.92–1.29)
>R$ 1500	217	166	0.76	Ref	Ref	196	111	0.57	Ref	Ref
Comorbidities										
Yes	290	241	0.83	1.11 (0.99–1.26)		261	164	0.63	1.13 (0.93–1.38)	
No	110	82	0.74	Ref		101	56	0.55	Ref	
Hypertension										
Yes	196	161	0.83	1.05 (0.95–1.15)		175	113	0.65	1.13 (0.96–1.33)	
No	204	162	0.79	Ref		187	107	0.57	Ref	
Diabetes										
Yes	113	100	0.88	1.14 (1.04–1.24)	1.09 (1.00–1.19)	98	69	0.70	1.23 (1.04–1.45)	1.15 (0.97–1.36)
No	287	223	0.78	Ref	Ref	264	151	0.57	Ref	Ref
Obesity										
Yes	57	50	0.88	1.10 (0.99–1.23)		54	40	0.74	1.27 (1.05–1.52)	1.22 (1.03–1.45)
No	343	273	0.80	Ref		308	180	0.58	Ref	Ref
Hypercholesterolemia										
Yes	43	40	0.93	1.17 (1.06–1.29)	1.15 (1.04–1.27)	38	26	0.68	1.14 (0.90–1.44)	
No	357	283	0.79	Ref	Ref	324	194	0.60	Ref	
Hypothyroidism										
Yes	30	28	0.93	1.17 (1.05–1.30)		27	20	0.74	1.24 (0.97–1.58)	
No	370	295	0.80	Ref		335	200	0.60	Ref	
Supplemental oxygen										
Yes	344	279	0.81	1.02 (0.87–1.18)		310	186	0.60	0.92 (0.73–1.14)	0.86 (0.70–1.06)
No	56	44	0.79	Ref		52	34	0.65	Ref	Ref
Length of hospital stay										
>8 days	198	168	0.85	1.10 (1.00–1.21)	1.08 (0.98–1.20)	176	112	0.64	1.10 (0.93–1.29)	
≤8 days	202	155	0.77	Ref	Ref	186	108	0.58	Ref	
Pronation										
Yes	130	105	0.81	1.00 (0.90–1.11)		118	77	0.65	1.12 (0.94–1.32)	1.15 (1.06–1.25)
No	270	217	0.81			244	142	0.58	Ref	Ref
Severity										
Mild	56	44	0.79	Ref		52	34	0.65	Ref	
Moderate	253	203	0.80	1.04 (0.89–1.20)		230	134	0.58	0.89 (0.71–1.12)	
Severe	91	75	0.83	1.08 (0.91–1.27)		80	52	0.65	0.99 (0.77–1.28)	

N: number of patients, PSC: post-COVID syndrome, PR: prevalance, PRR:prevalance ratio, aPRR: adjusted prevalance ratio, Ref: reference category; ^a^: calculated by Poisson regression with robust variance; ^b^: adjusted using forward stepwise regression selecting variables with a value of *p* < 0.20 in the univariable analysis. Age, sex, educational status, and individual income were retained irrespective of the *p*-value; ^c^: Adjusted using age, sex, educational status, individual income, diabetes, hypercholesterolemia, and length of hospital stay; ^d^: adjusted using age, sex, educational status, individual income, obesity, supplemental oxygen, and pronation; ^e^: follow-up loss of 38 patients.

## Data Availability

The data presented in this study are available upon request from the corresponding author.
